# Prediction of fruit characteristics of grafted plants of *Camellia oleifera* by deep neural networks

**DOI:** 10.1186/s13007-024-01145-y

**Published:** 2024-02-04

**Authors:** Fan Yang, Yuhuan Zhou, Jiayi Du, Kailiang Wang, Leyan Lv, Wei Long

**Affiliations:** 1 College of Computer and Information Engineering, Central South University of Forestry & Technology, Changsha, Hunan 410004 China; 2 Zhejiang Provincial Key Laboratory of Tree Breeding, Research Institute of Subtropical Forestry, Chinese Academy of Forestry, Hangzhou, Zhejiang 311400 China; 3 College of Hydraulic Engineering, Zhejiang Tongji Vocational College of Science and Technology, Hangzhou, Zhejiang 311231 China

**Keywords:** *Camellia Oleifera*, Grafting, Artificial neural network, Fruit characteristics

## Abstract

**Background:**

*Camellia oleifera*, an essential woody oil tree in China, propagates through grafting. However, in production, it has been found that the interaction between rootstocks and scions may affect fruit characteristics. Therefore, it is necessary to predict fruit characteristics after grafting to identify suitable rootstock types.

**Methods:**

This study used Deep Neural Network (DNN) methods to analyze the impact of 106 6-year-old grafting combinations on the characteristics of *C.oleifera*, including fruit and seed characteristics, and fatty acids. The prediction of characteristics changes after grafting was explored to provide technical support for the cultivation and screening of specialized rootstocks. After determining the unsaturated fat acids, palmitoleic acid C16:1, cis-11 eicosenoic acid C20:1, oleic acid C18:1, linoleic acid C18:2, linolenic acid C18:3, kernel oil content, fruit height, fruit diameter, fresh fruit weight, pericarp thickness, fresh seed weight, and the number of fresh seeds, the DNN method was used to calculate and analyze the model. The model was screened using the comprehensive evaluation index of Mean Absolute Error (MAPE), determinate correlation *R*^2^ and and time consumption.

**Results:**

When using 36 neurons in 3 hidden layers, the deep neural network model had a MAPE of less than or equal to 16.39% on the verification set and less than or equal to 13.40% on the test set. Compared with traditional machine learning methods such as support vector machines and random forests, the DNN method demonstrated more accurate predictions for fruit phenotypic characteristics, with MAPE improvement rates of 7.27 and 3.28 for the 12 characteristics on the test set and maximum *R*^2^ improvement values of 0.19 and 0.33. In conclusion, the DNN method developed in this study can effectively predict the oil content and fruit phenotypic characteristics of *C. oleifera*, providing a valuable tool for predicting the impact of grafting combinations on the fruit of *C. oleifera*.

**Supplementary Information:**

The online version contains supplementary material available at 10.1186/s13007-024-01145-y.

## Introduction

*C.oleifera*, an evergreen shrub or small tree in the Camellia of the Theaceae, is an essential woody edible oil tree in southern China with a cultivation history of over 2000 years [1]. It is an edible oil obtained by pressing the ripe seeds of *C.oleifera*. With a unique woody oil in China, it has been known as a treasure in oil since ancient times [2]. The unsaturated fatty acids, including oleic acid, linoleic acid, and linolenic acid in the seed oil of *C.oleifera*, are up to 83%~95%, ranking high among all edible vegetable oils [3–5]. Grafting is the primary means of breeding and improving *C.oleifera* varieties in high-quality, high-yield cultivation. Adopting grafting seedling cultivation technology can not only fully leverage the advantages of rootstock varieties, improve the quality of scion varieties, and increase the yield of *C.oleifera* trees, but also expand the planting range of *C.oleifera*, reduce planting costs, and increase economic benefits [6–8].

Since the interaction between rootstock and scion is a common phenomenon in plant grafting, the rootstock affects the flowering, fruiting, and traits of the scion through gene exchange [9–13]. These changes may be beneficial for improving fruit quality but can also have an adverse effect. It has an impact on the stability of the product. Research has found a significant rootstock and scion interaction in the grafted seedlings of *C.oleifera* [14–17], and scions control root growth after grafting [14], and the effect of scions on nutrient absorption [18]. There are significant differences in tree potential and growth among different varieties [19]. Therefore, for *C.oleifera*, which is mainly propagated by grafting, obtaining stable fruit characteristics and making grafting beneficial for fruit stability are issues of interest. Nowadays, changes in fruit characteristics after grafting under the influence of the interaction mechanism between rootstock and scion are commonly used based on long-term observation and measurement analysis to screen for rootstocks with high affinity that are beneficial for improving tree or fruit quality [20, 21]. This process costs more time and human resources. It is not conducive to the rapid promotion and application of high-quality varieties. Thus, there is a need for a prediction fruit characteristics technology after grafting for early selection in the rootstock so that it can reduce the rootstock’s effect.

There have been many significant achievements in AI research in agriculture. It has made significant progress in the Internet of Things control, pest control, variety identification, and yield prediction, especially in wheat, corn, and rice [22, 23]. However, in the complex growing environment of forestry, the diversity of topographical and climatic environments makes the study and application of forestry still in forest resource investigation. At the same time, the vast market demand for *C.oleifera*, China’s most promising woody oil tree, and the supply-demand contradiction between production and demand are pressing for rapid improvement in quantity and quality. Predicting growth and yield is currently one of the most challenging problems in precision agriculture, and many models have been proposed and validated so far. This issue requires multiple datasets, as growth and yield depend on many factors, such as climate, weather, soil, fertilizer use, and seed variety [24]. This indicates that predicting growth and yield is a challenging task. Today, predictive models can reasonably estimate growth and yield, but better yield prediction performance is still needed. The rational and efficient use of AI to promote the development of the *C.oleifera* industry is of great significance at both economic and strategic levels.Traditional machine learning (ML) methods, such as decision trees, naive Bayesian algorithms, fuzzy logic, support vector machines, and gradient enhancement algorithms, typically require manual participation in feature extraction and preprocessing steps before model use [25]. Handcrafted feature extraction and non-standard preprocessing measures limit model scalability, making the analysis time-consuming and challenging. Experts with sufficient knowledge are always necessary and considered critical [26]. Support Vector Machine (SVM) is widely used in research fields such as data classification and prediction [27–31] due to its effectiveness in machine learning and reliance on structural risk minimization. In addition to classification and prediction, Support Vector Regression (SVR) is another application of SVM that specifically addresses regression problems. For example, Guo et al. introduced an active multi-classification method based on SVM [32]. Furthermore, it successfully distinguished the level of moldiness in corn granules using SPA and SVM [33]. Random forests are based on the concept of Bagging. Random forest adds a new feature to Bagging: randomly choosing a random number of features and constructing a tree with them, repeating this procedure many times, and continuously averaging all the predictions made by all trees [34]. Therefore, random forests have processed errors’ bias and variance components and have been proven robust [35]. Currently, RF algorithms have been applied to agricultural research. For example, sugarcane yield was estimated by RF algorithm according to different prediction ranges and achieved good results [36]. It also established a rice yield estimation model and conducted model precision evaluation [37].

**Fig. 1:**
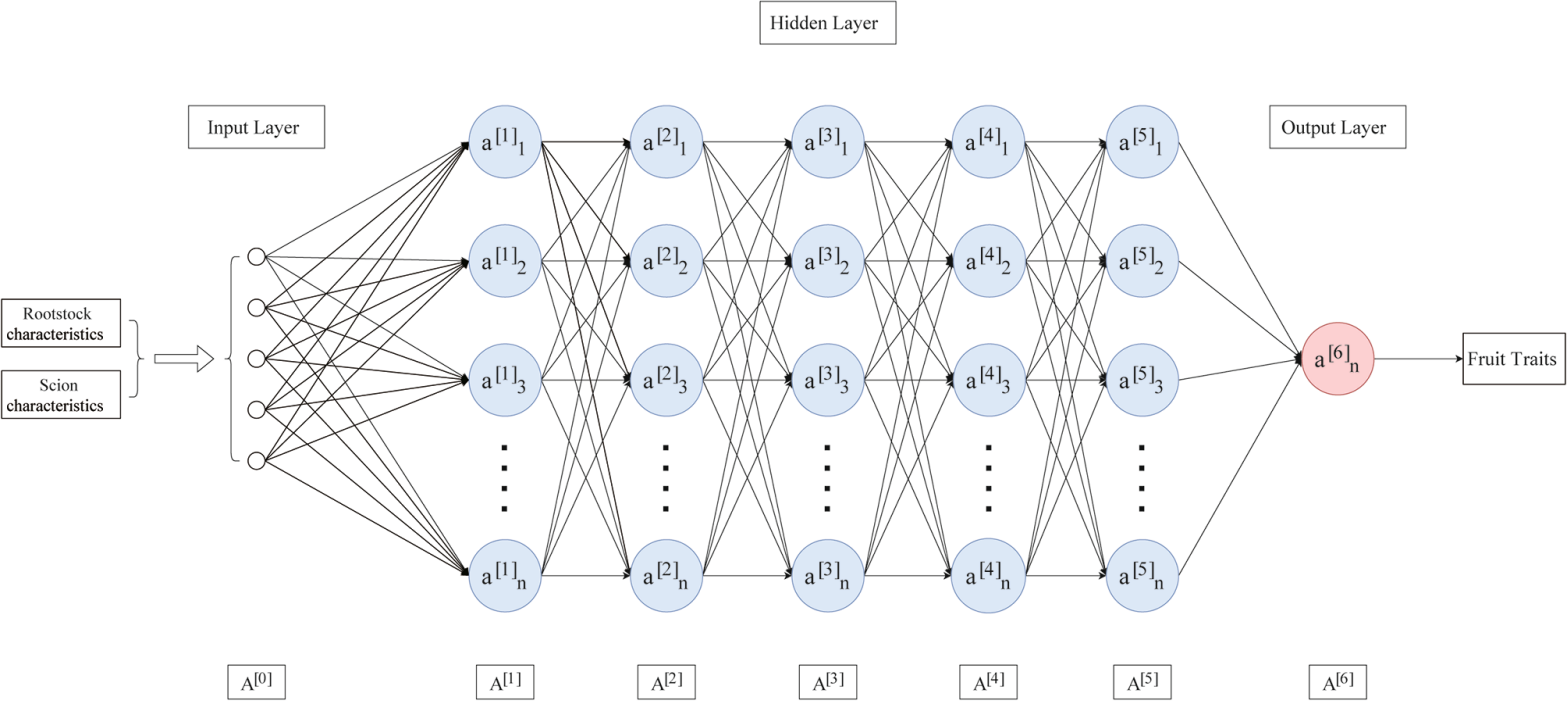
The structure of DNN modle

Over the past decade, neural networks have covered almost all scientific fields and become an essential ingredient for various real-world applications. Deep neural networks (DNN) have multiple characteristics of nonlinear mapping, which can fit highly complex functions. It can autonomously learn and find relevant information between them and continuously improve the optimization results. It has become an indispensable tool in various applications such as image classification, speech recognition, or natural language processing. These techniques have achieved high prediction accuracy, and in many cases, they are comparable to human performance. Due to the continuous development of modern computational methods, data-based prediction methods are increasingly being applied in various fields [38, 39]. This method provides fast and accurate results for agricultural applications, such as predicting greenhouses’ internal temperature or transpiration rate [40, 41]. It used deep learning techniques such as convolutional neural networks and recurrent neural networks to predict soybean yield in the United States based on a sequence of remotely sensed images taken before the harvest [42, 43]. Their model outperformed traditional remote-sensing-based methods by 15% regarding Mean Absolute Percentage Error (MAPE). The convolutional neural networks were used to predict crop yield based on satellite images [44]. Their model uses 3D convolutions to include spatiotemporal features and outperforms other machine learning methods. Khaki and Wang et al. designed a deep neural network model (Fig. 1) for predicting corn yields at 2247 locations from 2008 to 2016 [45]. Wang et al. designed a deep learning framework to predict soybean crop yields in Argentina, and they also achieved satisfactory results by using transfer learning methods to predict soybean yields in Brazil with less data [46]. The key to a deep neural network model is that it does not require the specification of appropriate functions to fit the relationships between the data. It can learn and find the relevant information between them autonomously and continuously improve the optimization results. The DNN also provides a general approximation framework, meaning that no matter what function we want to learn, the deep neural network can represent such a function [47, 48]. Deep neural networks belong to the class of representation learning models that can find the underlying representation of data without handcrafted input of features and have multiple stacked nonlinear layers that transform the raw input data into higher and more abstract representations at each stacked layer [49]. As the network grows more profound, more complex features are extracted, contributing to the results’ higher accuracy. Given the suitable parameters, DNNs are known to be universal approximation functions, meaning they can approximate almost any function, although finding suitable parameters may be challenging [50, 51].

Compared with the aforementioned neural network models in the literature, which were shallow networks with a single hidden layer, deep neural networks with multiple hidden layers are more potent in revealing the fundamental nonlinear relationship between input and response variables [49], but they also require more advanced hardware and optimization techniques to train. For example, the neural network’s depth (number of hidden layers) significantly impacts its performance. Increasing the number of hidden layers may reduce the classification or regression errors. Still, it may also cause the vanishing/exploding gradients problem that prevents the convergence of the neural networks [45, 52–55].

In summary, the success of DNNs in solving problems depends on several factors, including the training data’s size, type, quality, and preprocessing steps. What sets DNNs apart is their ability to learn and discover correlations between data autonomously, without the need to specify appropriate functions to fit the relationships. Deep neural networks can represent any function we want to know, providing an accurate framework for predicting various indicators in *C.oleifera* grafting assemblages. This will enable practical data analysis and technical guidance for *C.oleifera* grafting. In this study, we wish to obtain a robust DNN algorithm predictive model that can quickly and accurately predict the oil content and phenotypic characteristics of *C.oleifera* fruit bodies to help us predict in advance the effect of grafting combinations on fruit properties. I have provided practical data analysis and technical guidance for *C.oleifera* grafting.

## Materials and methods

### Materials

We conducted a study in Guanshang Town, Zhangshu City, Jiangxi Province, China, collecting fruits from trees over 6-year-old of 12 varieties, including CL3, CL4, CL18, CL23, CL27, CL40, CL53, CL166, etc. 106 grafting combinations were obtained by scions and half-sibling seed rootstocks of 12 varieties (Table S1 and Table S2). The fruits of these tree combinations were collected for the determination of characteristics. Five trees, each of similar age, good growth, and free from pests or disease, were selected for each combination. We randomly selected and measured 30 fruits from each combination. After peeling and drying the seeds, we proceed to the subsequent measurement. Each test consisted of three biological replicas. All samples were collected by institutional, national, or international guidelines and legislation. The local forestry management authority authorized the collection of all samples for this study.

### Determiation of Fruit characteristics

To measure the characteristics of fruit, including the weight (g), height (mm), and diameter (mm) of fresh fruits, the weight of dried seeds (g), dried kernels (g), and kernel oil content (g), we can use a vernier caliper with a sensitivity of 0.01 mm and a 0.01 g electronic balance. Additionally, we can calculate the fruit shape index, kernel ratio of dried seeds, oil content of dried kernels ratio, and dry seed oil content using specific formulas. The fruit shape index is calculated by dividing the fresh fruit height by the fresh fruit diameter and multiplying the result by 100%. The oil content of dried kernels can be calculated by dividing the weight of kernel oil by the weight of dried kernels, multiplied by 100%. The dry kernel oil ratio can be calculated by dividing the weight of kernel oil by the weight of dry seeds, multiplied by 100%. The dry seed oil ratio is calculated by multiplying the kernel oil content by the kernel-fruit ratio of dry seeds and multiplying the result by 100%.

### The oil extraction from seeds by Soxhlet extraction (SE)

All samples of *C. oleifera* seeds were powdered by a laboratory plant grinder. Approximately 10 g of ground sample were weighed and recorded as w0 (g), then transferred to a Soxhlet extractor filled with 180 mL petroleum ether (60–90 ℃), and extracted at 88 ℃ for 6 h. Finally, the solvent was evaporated under vacuum. The residual was dried at 60 C in a vacuum to a constant weight of w1(g). The oil content is calculated and expressed by the formula: w = w1/w0 100%. Experiments were carried out on three biological replicas.

### Fat analysis of the extracted oil of *C. Oleifera* by GC

As FA of *C. oleifera* oil presented in the form of fatty acid triglycerides in general, it must be transformed to be methyl esters of fatty acids means of sodium hydroxide. 0.2 ml of the extracted *C. oleifera* oil were put in 10 mL tube. Two millilitres of 0.5 mol/L sodium hydroxide–methanol was added into the tube, shook, and then placed at 60 ℃ in water-bath for 30 min, 5 mL nhexane were added. The supernatant was taken for injection to a gas chromatography spectrometer (HP6890 series, Agilent Techologies Inc.), equipped with a Hp-5 capillary column (30 m 0.25 mm 0.25 lm). The injector and detector temperature were set at 280 ℃. The oven temperature was programmed from 100 ℃ to 270 ℃ with a speed of 5 ℃/min and a final hold of 5 min. The signals from the detector were integrated as normalised percentages from the calibration curve by the HP software, and the main four individual fatty acid (oleic, linoleic, palmitic, stearic acid) were expressed as % of the total fatty acids. The unsaturated acids were considered as the sum of the oleic acid and linoleic acid.

### Deep neural network (DNN)

This study used the combination of rootstock and scion varieties as input. It measured the parameters of *C.oleifera* outputs, including y1: Palmitoleic acid C16:1, y2: cis-11 eicosane acid C20:1, y3: unsaturated fatty acid, y4: oleic acid C18:1, y5: linoleic acid C18:2, y6: linolenic acid C18:3, y7: oil content, y8: fruit height, y9: fruit diameter, y10: fresh fruit weight, y11: pericarp thickness, y12: fresh seed weight, respectively. Since there were significant variations in fruit phenotypic characteristics among different varieties, five parameters, namely fruit height, fruit diameter, fresh fruit weight, fresh seed weight, and number of fresh seeds, were selected instead of the variety number. The hidden layer between the input and output layers can consist of one or more layers. The number of layers and neurons depends on the number of samples and the complexity of the task. Generally, a deeper and more layered model can improve accuracy by providing better nonlinear expression ability. This enables the model to learn complex transformations and adapt to more complex feature inputs. However, more network parameters also require more time and samples for training.

We collected 30 fruits from each grafting combination, measured their characteristics, and took the average as a sample. Due to missing data in the two combinations, to ensure the authenticity and consistency of the data, we cleaned and sorted the data, resulting in datasets of 106 valid samples. Of the datasets in 106 samples, 74 are used for training and 21 for validation, while the rest are used for testing. Using the Relu activation function in the hidden layer is necessary for the network to learn nonlinear functions. The output layer uses the linear transfer function directly, and each hidden layer is connected with a dropout function (with a dropout rate of 0.1) to temporarily discard network information and reduce overfitting. The DNN uses the Adaptive Moment Estimation(Adam) optimizer for training (200 epochs), with a learning rate of 0.01. The loss function selects the Mean Squared Error (MSE), while the evaluation index selects the Mean Absolute Percentage Error (MAPE) to measure the performance of the model. The model that performed the best was selected based on the MAPE values of the validation set, and comprised 5 fully connected layers (1–5) and 8 neurons (2, 4, 8, 16, 32, 64, 128, 256) with different numbers.

1 $${\text{MSE}}=\frac{1}{{\text{m}}}\mathop \sum \limits_{{{\text{i}}=1}}^{{\text{m}}} {({y_{\text{i}}} - {\hat {y}_{\text{i}}})^2}$$

m: the number of input samples, ${\text{y}}_{\text{i}}$: the true value of the sample, ${\widehat{\text{y}}}_{\text{i}}$: the predicted value of the sample.

2 $${\text{MAPE}} = \frac{{100\% }}{{\text{n}}}\sum\limits_{i = 1}^n {\left| {\frac{{{{\hat y}_i} - {y_i}}}{{{y_i}}}} \right|}$$

After the model is established, the prediction accuracy of the dependent variable is evaluated by calculating the correlation coefficient, which is calculated when comparing the actual value with the predicted value. The determination (R2) is one of the most commonly used methods, independent of the model, used to evaluate the statistical parameters of the developed model (Eq. (3)).

$${R^2}=\frac{{\sum\nolimits_{{i=1}}^{n} {{{\left( {\hat {y} - y} \right)}^2}} }}{{\sum\nolimits_{{i=1}}^{n} {{{\left( {y - \bar {y}} \right)}^2}} }}$$

*ŷ*: prediction, *y*: true, $\bar y$: the average of the true, *n*: number of samples.

This article constructs a double loop that combines the number of hidden layer layers and the number of neurons to form a network model. The training and validation sets are inputted to obtain the minimum MAPE value in each epoch and store the corresponding model information.

### Data analysis

The experimental data were organized and analyzed using PyCharm 2020, Anaconda 3, and Tensorflow 2.1. The regression equation were analyzed and plotted using GraphPad 8.4.

## Results

### Characteristics of *C.oleifera* under different grafting combinations

*C.oleifera* is a valuable oil crop with a wide range of applications. To study the effect of different rootstock and scion combinations on the quality of *C.oleifera*, we conducted a comprehensive evaluation by taking into account the following parameters: Palmitoleic acid C16:1, cis-11 eicosane acid C20:1, unsaturated fatty acids, oleic acid C18:1, linoleic acid C18:2, linolenic acid C18:3, kernel oil content, fruit height, fruit diameter, fresh fruit weight, pericarp thickness, and fresh seed weight. We found that the fruit varieties in *C.oleifera* significantly differ under different rootstock and scion combinations (Table S1 and Table S2). Different combinations of rootstocks and scions can significantly affect the fruit characteristics of *C.oleifera*. When CL18, CL40, and CL53 are scions, there are differences in fruit characteristics among different grafting combinations. Grafting CL18 onto the half-sib rootstock of CL22, CL21, and CL27 significantly increased the height and diameter of the fruit, with significant differences compared to the original rootstock. Grafting with CL26 and CL40 half-sib rootstocks resulted in a lower pericarp thickness while grafting with CL27 had the highest thickness. The CL40 had the highest grafting consequences on the stock, while the fruit diameter was highest after grafting on the CL21 half-sib rootstock. It has the highest grafting consequences and minor fruit diameter compared to the CL4 half-sib rootstock and is significantly different from the other combinations. After grafting with CL59 and CL4 half-sib rootstocks, the thickness of the pericarp is lower. CL53 has the highest fruit height and diameter after grafting with CL3 half-sib rootstock during scion, while it has the lowest after grafting with CL21 half-sib rootstock, which is entirely different from the performance of CL26 as rootstocks. When CL59 and CL40 are rootstocks, the fruit pericarp thickness after grafting is the lowest, while it is the highest after grafting with CL21 half-sibling rootstock.

As an essential characteristics in the production value of *C.oleifera*, the content of oil and fatty composition were found to differ among grafted varieties in this study, and rootstocks may impact the content of oil and fatty composition (Table S2). CL18 reaches its highest value at CL26 half-sibling rootstock, followed by CL59 half-sibling rootstock, while this stock has the lowest value, showing significant differences after grafting with multiple stock varieties. When the CL40 is grafted onto the CL27 half-sibling rootstock, it reaches the highest, followed by the CL59, and the CL21 and CL40 half-sibling rootstock are the lowest. The CL21 and CL27 half-sibling rootstocks are also lower among the CL53 half-sibling rootstock combinations, while the CL59 half-sibling rootstock is the highest. The CL59 half-sibling rootstock may affect the improved oil content. In addition, the content of oleic acid is an essential indicator for evaluating the quality of the oil of *C.oleifera*, and grafted oleic acid exhibits differentiated performance. When CL59 is used as the rootstock, there is a significant proportion of oleic acid content among various combinations. When the CL21 half-sibling rootstock was used as the rootstock, there was a decreasing trend in the oleic acid content of CL18 and CL40, whereas CL53 showed a significant increase. From these two indicators, the CL59 half-sibling rootstock may have a significant regulatory effect on the oil content and oleic acid content. Furthermore, it can also be seen that some varieties of rootstocks have different effects on the oil composition and fruit characteristics.

### The MAPE values of fruit characteristics for different grafting combinations

**Fig. 2:**
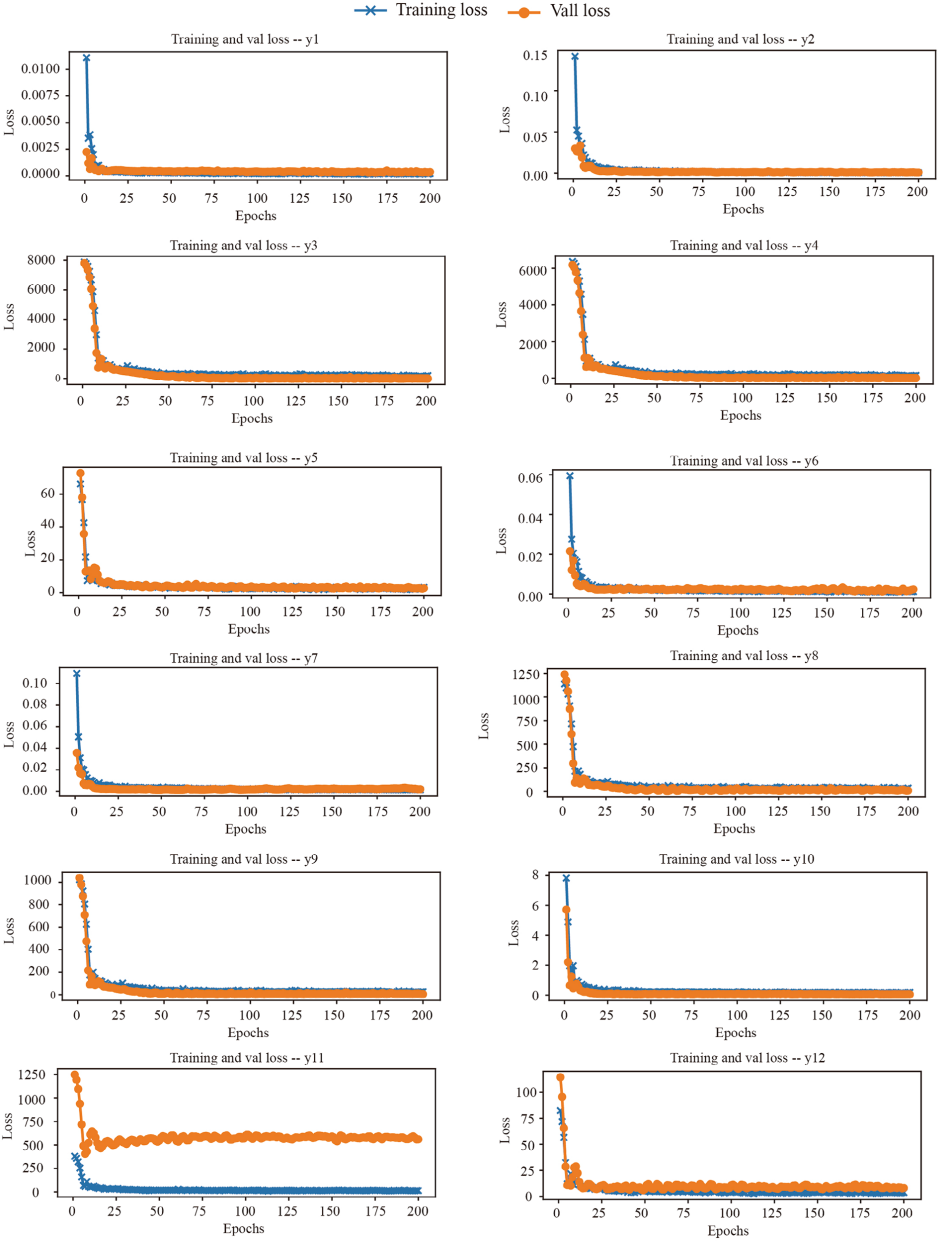
Evaluation of changes in loss plot across 200 epochs of training and testing datasets with 12 fruit characteristics. yl: palmitoleic acid C16:1, y2: cis-11 eicosanoic acid C20:1, y3: unsaturated fatty acid, y4: leic acid C18:1, y5: linoleic acid C18:2, y6: linolenic acid C18:3, y7: kernel oil content, y8: fruit height, y9: fruit diameter, y10: fruit weight, yll: pericarp thickness, y12: fresh seed weight

After data segmentation and processing, 40 model combinations were constructed, ranging from 2 neurons in 1 hidden layer to 256 neurons in 5 hidden layers. These combinations were used to construct different levels of DNN, train the network, and predict 12 phenotypic characteristics to obtain the minimum MAPE value for different combinations in the DNN model under 12 characteristics (Table S3). When the MAPE value is greater than 25, the difference between the predicted and actual values is too significant, and the prediction is not accurate, so the data are discarded. Although it is impossible to achieve the optimal performance of the model on each feature, observing the performance of the model on the 12 characteristics by ranking the average of the 12 characteristics helps to evaluate the overall performance of the model. To evaluate the performance of the DNN model in predicting 12 fruit characteristics, we ranked all MAPE values by taking the average. It was found that the average values obtained from 3 hidden layers and 16 neurons were the lowest (Table S3). So, it was selected as the optimal model. Then, during the training and validation of the training and validation sets, both the train loss and val loss values gradually decrease with the increase of epoch, and the loss values of each characteristic gradually decrease. After reaching the bottom, it gradually stabilizes (Fig. 2), indicating excellent convergence on both the training and validation sets.

### Prediction results of fruit performance characteristics under different model combinations

**Fig. 3:**
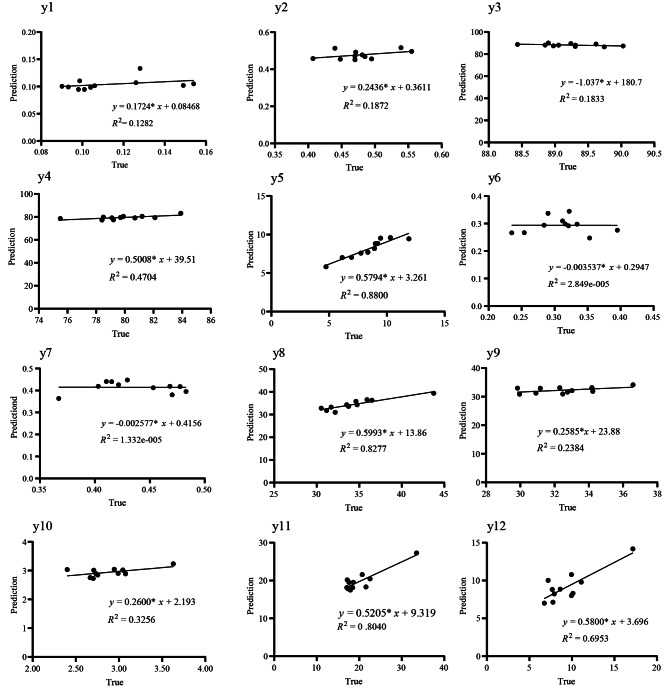
Actual and predicted values by regression validation with *R*^2^. yl:Palrnitoleic acid C16:1; y2: cis-11 eic-osane acid C20:1; y3: unsaturated fatty acid; y4: oleic acid C18:1; y5: linoleic acid C18:2; y6: linolenic acid C18: 3; y7: kernel oil content y8: fruit height; y9: fruit diameter; y10: fresh fruit weight; y11: pericarp thickness; y12: fresh seed weight

To further verify the model’s accuracy, linear regression equation analysis was performed on the predicted and measured values of the test and validation sets (Fig. 3). Overall, the predicted and measured values fit well together. However, there are differences in determination (*R*^2^) between characteristics, such as *R*^2^ values below 0.1 for y6 and y7. The *R*^2^ remains high, reaching a maximum of 0.88, including y5, y8, y11, y12, y4, and y10. Interestingly, these characteristics are critical to *C.oleifera* as a wood oil tree. The changes in kernel oil content of woody oil tree such as olive and oil palm are often predicted by visual methods such as image and spectrum combined with algorithms such as ANN, DNN, and CNN in different cultivation environments [56–60]. However, there are few reports on predicting fruit characterisrtics after grafting based on genetic characteristics, especially when the oil characteristics are mainly quantitative genetic characteristics. So, the DNN algorithm could predict the characteristics of the fruit early after grafting and understand the impact of differentiated rootstock on the fruit of the scion, indicating that the model has a high value in predicting the fruit characteristics of *C.oleifera*.

### Comparison results with support regression vector (SVR) and random forest (RF) models, DNN

The performance difference between traditional machine learning methods and DNN models in predicting phenotypic characteristics of fruits is further compared (Table S4). The same training and validation sets were run on SVR and RF models to obtain MAPE and *R*^2^ values (Table S5 and Table S6) and compare them with the results of DNN models. Among the 12 fruit characteristics, the MAPE value of DNN compared to SVR can be reduced by up to 7.27, the *R*^2^ value can be increased by up to 0.19, the MAPE value of DNN compared to RF can be reduced by up to 3.28, and the *R*^2^ value can be increased by up to 0.33 (Table 1). Compared to the SVR and RF models, the DNN is able to reduce the error, especially for y2, which significantly improves the test set. Of particular note, the DNN model has shown significant advantages in predicting y11, with an improvement of 0.14 compared to the SVR model and 0.56 compared to the RF model (Table 1).

## Discussion

### Regulatory effect by grafting on the fruit characteristics and oil content of *C.oleifera*

Grafting is an ancient plant reproduction technique where the scion and rootstock are grafted and healed to form a new plant [61, 62]. Rootstock plays an essential role in affecting scion growth [11, 63], growth, development, yield, and potential flowering and fruit quality by releasing or improving the absorption and transportation of mineral nutrients, hormones, and carbohydrates, thereby affecting the increase in fruit yield, quality, and quality [64,65,66]. Therefore, the choice of appropriate rootstock is an important determining factor for achieving high and stable fruit performance. This study observed differences in fruit characteristics, oil content, and fatty acid composition among scions of the same variety under the action of differentiated rootstocks after grafting. Of particular concern is that the half-sib rootstock of CL21 and CL59 significantly affects the characteristics of each variety. This indicates that using only species as the selection criteria for oil tea rootstocks may pose a risk to yield quality after oil tea grafting. At the same time, it also indicates that the interaction between rootstocks and scions in *C.oleifera* is not only the regulation of root growth by scions [67,68], but also reflected in the influence of fruit characteristics. Due to the significant impact of rootstocks on the physiological characteristics and other aspects of the growth and development of grafted plants, the reduction of scion growth is one of the most interesting phenomena. Therefore, it is necessary to explore the critical mechanisms of regulating fruit characteristics between rootstocks and scions and understand the role of hormones and other substances in the development of the entire post-grafting period.

### The quality of fruit through characteristics data prediction after grafting achieves early selection of rootstock

Deep neural networks belong to phenotype learning models with multiple stacked nonlinear layers that transform the raw input data into higher and more abstract representations for each stacked layer [69]. Enabling it to extract more complex features as the network deepens can help improve the accuracy of the prediction. Therefore, it is widely used to predict crop yield and fruit characteristics in many plants, including corn yield [70–73], firmness, soluble solids content (SSC) and growth characteristics of apple [73, 74], the volume of carrot and apple [75, 76], classification in bananas [77], the incidence of the blister moth in leaves of apple [78], stress response in orange [79]. These studies have demonstrated the effectiveness and reliability of deep learning models. This study uses a DNN model to predict 12 personality characteristics of fruits. Under the optimal model, the MAPE of fruit pericarp thickness (y11) was the highest on the validation set, at 16.39, while the MAPE of unsaturated fatty acids (y3) was the lowest, at 2.38 (Table S3), showing a significant difference. It is commonly believed that the higher the correlation, the lower the MAPE value and the more accurate the prediction. This may be due to the different correlations between the input and predicted features, which is also a factor for the difference in MAPE. In addition, if the actual and predicted values perform nicely in regression for certain fruit characteristics, the values range from 0 to 1. The positive value of *R*^2^ can be considered similar to the accuracy obtained by regression [80]. In this study, all characteristics have *R*^2^ values between 0 and 1, but y6 and y7 have relatively low values. This may be due to the small range of valid values (Table S4), which resulted in a small proportion of prediction error to actual values. However, the correlation between predicted and actual values was low, affecting the prediction results (Table S4).

It should be noted that the *R*^2^ values for key fruit characteristics that affect the yield of woody oil trees, including fruit height, fruit weight, fresh seed weight, and oil quality oleic and linoleic acid content, are relatively high. In contrast, the MAPE values are still relatively low. This suggests that DNN can be used for the early selection of rootstock before grafting, reducing the impact of rootstock and scion interactions on fruit characteristics after grafting.

### The prediction of fruits in *C.oleifera* can be better achieved with DNN

SVR and RF, as traditional machine learning methods, have been widely applied to estimate production, environmental changes, and other factors. Data-driven machine learning methods have shown great potential in parameter estimation. Deep learning algorithms such as DNN have quickly become the primary method for predicting feature extraction in recent years. These three methods have been widely used in many studies, such as remote sensing classification [81, 82], landslide monitoring [83], and drought monitoring [84]. In recent years, there have been studies using SVR, RF, and DNN for crop yield prediction [42, 85]. This study evaluated the performance of SVR, RF, and DNN models by *R*^2^ and MAPE. Among the three algorithms compared, the DNN algorithm showed an improvement in MAPE values compared to SVR and RF (Table 1), indicating the advantages of using DNN for prediction.

Generally, in fruit characteristics prediction, obtaining a large amount of actual fruit data was difficult, and it took a lot of labor, resource, and time to collect sample data in the field. Therefore, extracting effective characteristics from limited samples is particularly important. The DNN model can handle nonlinear datasets and has a certain tolerance for noise and interference. It can also achieve complex feature transformations through multi-layer neural networks and activation functions without the need for tedious feature engineering, which is beyond the capabilities of SVR and RF [86]. In this study, by reasonably setting the number of layers, number of neurons, optimization function, dropout layer, activation function, and iteration number of DNN, DNN surpassed RF in 9 features (y1, y2, y5, y6, y7, y8, y10, y11, y12) and SVR in 7 characteristics (y1, y2, y3, y5, y8, y11, y12). SVR outperformed RF in 7 characteristics (y4, y5, y6, y7, y8, y9, y10). SVR used the inner product kernel function instead of nonlinear mapping to high-dimensional space. A few support vectors determined the final result, which not only helped to seize the key samples and remove a large number of redundant samples but also showed that the algorithm was simple and had good “robustness.” Therefore, the performance of SVR was second only to DNN (Fig. 3; Table 1). RF might lead to over-fitting when there is limited training data. Although the RF might overfit when training small sample data, it was an integrated algorithm that could effectively enhance the performance of a single classifier [87]. Generally, RF could achieve higher accuracy and lower variance and deviation to produce more satisfactory results [88]. Therefore, the performance of RF was only slightly worse than that of DNN and SVR (Table 1). This study’s SVR, RF, and DNN models could produce acceptable results for fruit characteristics prediction (Table 1). This article further compared the results of using SVR, RF, and DNN with the results of other studies. Ang et al. (2020) used DNN comparing with SVR, RF, and accuracy between oil palm yield and actual yield [89]. After backward elimination, the DNN achieved the highest prediction accuracy among the other models, with a 14% increase in *R*^2^ and a 1% decrease in MAPE. In this study, critical characteristics such as oleic acid, linoleic acid, fruit height, and fruit weight in fruits of *C.oleifera* have more declining MAPE values and increasing *R*^2^ in the DNN. Therefore, this paper’s prediction of fruit characteristics results was reliable. Interestingly, although the kernel oil content of fruit (y7) has a lower MAPE value, the *R*^2^ value is lower. This may be related to the influence of genetic characteristics and environmental factors on kernel oil content.

## Conclusion

This study focused on *C.oleifera* and found differences in fruit characteristics between the same variety and different rootstocks after grafting. Therefore, the research uses pre and post-grafting fruit phenotype data to establish a model for predicting fruit characteristics using deep neural networks.By setting different levels of hidden layers and the number of neurons, it was found that when using 3 hidden layers and 16 neurons, the overall performance achieved the best. The MAPE values of this model on the test set are 0-17.69. Compared to the traditional SVR and RF models, the DNN achieves a MAPE improvement rate of 7.27 and 3.28 for the 12 characteristics on the test set and a maximum *R*^2^ improvement value of 0.19 and 0.33, which is better than the SVR and RF models. It indicates that the DNN model is more accurate and stable, avoiding traditional machine learning model selection. They can predict the phenotypic characteristics of fruit after grafting with *C.oleifera*. This achievement can provide adequate technical support for improving the cultivation of tung oil trees. In addition, accurate prediction and evaluation systems can be developed by adding more *C.oleifera* varieties, enriching fruit prediction parameters and input characteristics, improving model accuracy, and other means to help determine the impact of variety grafting on fruit characteristics, thereby reducing the time and labor costs of related experiments.

**Table 1:** Comparison of MAPE value improvement rates among DNN, SVR and RF models under 12 characteristics

model		y1	y2	y3	y4	y5	y6	y7	y8	y9	y10
RF	MAPE	-1.30	-0.49	1.09	0.27	-3.28	0.36	1.44	0.18	0.53	-1.55
	*R* ^2^	0.13	0.19	-0.27	-0.25	0.21	0.00	0.00	0.05	-0.13	0.33
SVR	MAPE	-7.27	-3.03	1.04	0.68	0.78	-0.18	0.69	-1.20	0.77	0.33
	*R* ^2^	0.13	0.19	0.17	-0.33	0.01	-0.10	-0.22	0.05	-0.28	-0.09

* Positive values as an increase, and negative values (-) a decline. y1:Palmitoleic acid C16:1; y2: cis-11 eicosane acid C20:1; y3: unsaturated fatty acid; y4: oleic acid C18:1; y5: linoleic acid C18:2; y6: linolenic acid C18:3; y7: kernel oil content; y8: fruit height; y9: fruit diameter; y10: fresh fruit weight; y11: pericarp thickness; y12: fresh seed weight, respectively

### Electronic supplementary material

Below is the link to the electronic supplementary material.

Additional file 1:Table S1 Analysis of variance in fruit characteristics under different grafting combinations in *Camellia oleifera*

Additional file 2: Table S2 Analysis of variance in fat and fatty acid composition of fruit under different grafting combinations in *Camellia oleifera*

Additional file 3: Table S3 The MAPE values of 12 characteristics of fruits under different combinations

Additional file 4: Table S4 The MAPE values and *R*^2^ based on the validation and testing sets of the 12 characteristics in the DNN mode

Additional file 5: Table S5 The MAPE values and *R*^2^ based on the validation and testing sets of the 12 characteristics in the RF mode

Additional file 6: Table S6 The MAPE values and *R*^2^ based on the validation and testing sets of the 12 characteristics in the SVR modes

## Data Availability

The datasets used and/or analyzed in the current study are available from the corresponding author upon reasonable request.

## Declarations

### Permission to use varieties

The varieties owner has approved us to use 12 varieties of *Camellia oleifera* in current research. The local forestry management department authorizes the collection of all samples for this research.
